# Differential consequences of obstetric complications and childhood maltreatment on neurodevelopmental, emotional and behavioral outcomes in children and adolescents: exploring sex differences

**DOI:** 10.1192/j.eurpsy.2026.10552

**Published:** 2026-07-31

**Authors:** N. San Martín-González, L. Marqués-Feixa, S. Romero, J. March-Llanes, M. Rapado-Castro, I. Zorrilla, H. Blasco-Fontecilla, S. Papiol, L. Fañanás

**Affiliations:** 1 Department of Evolutionary Biology, Ecology and Environmental Sciences, University of Barcelona, Barcelona; 2 Centre for Biomedical Research Network on Mental Health (CIBERSAM), Madrid; 3 Biomedicine Institute of the University of Barcelona (IBUB); 4 Department of Child and Adolescent Psychiatry and Psychology, Institute of Neuroscience, Hospital Clínic de Barcelona; 5 Institut d’Investigacions Biomediques August Pi i Sunyer (IDIBAPS), Barcelona; 6 Department of Psychology, Faculty of Education, Psychology and Social Work, University of Lleida, Lleida; 7 Department of Child and Adolescent Psychiatry, Institute of Psychiatry and Mental Health, Hospital General Universitario Gregorio Marañón; 8 School of Medicine, Universidad Complutense, IiSGM, Madrid, Spain; 9 Department of Psychiatry, Melbourne Neuropsychiatry Centre, The University of Melbourne & Melbourne Health, Victoria, Australia; 10 Department of Psychiatry, Hospital Santiago Apostol, Vitoria; 11 Department of Psychiatry, Puerta de Hierro University Hospital-Majadahonda , ITA Mental Health, Madrid; 12 Instituto de Investigación, Transferencia e Innovación, Ciencias de la Salud y Escuela de Doctorado, Universidad Internacional de La Rioja, Logroño, Spain; 13 Institute of Psychiatric Phenomics and Genomics (IPPG), University Hospital, LMU Munich; 14 Department Clinical Translation, Max Planck Institute of Psychiatry, Munich, Germany

## Abstract

**Introduction:**

Early-life adversity, including pre/perinatal stress and childhood maltreatment (CM), is strongly linked to neurodevelopmental and psychiatric outcomes. However, the specific impact of these stressors and the differential effects in males and females remain poorly understood.

**Objectives:**

This study aims to investigate the relative and combined impact of maternal psychosocial stress during pregnancy, obstetric complications (OC), and CM on neurodevelopmental delays, emotional/behavioral symptoms, and DSM-5 diagnoses before puberty (12 y.o.), with a focus on sex differences.

**Methods:**

211 participants aged 7–17 from the Spanish multicenter Epi_Young_Stress study were included (130 with psychiatric diagnoses and 81 controls). 26.06% of children presented a psychiatric diagnosis and 25.1% a neurodevelopmental disorder before puberty. Hierarchical and logistic regression models were used. Neurodevelopmental subdomains, CBCL dimensions, overall adjustment (CGAS), and DSM-5 diagnoses were the studied outcomes. Predictor variables included socioeconomic status, maternal distress during pregnancy, OC, CM, and IQ. Interaction effects between prenatal and postnatal stressors were tested.

**Results:**

OC were associated with motor and language neurodevelopmental delays and attentional problems and worst adjustment only in males. CM emerged as the most consistent and robust predictor of emotional/behavioral problems, worst overall adjustment and psychiatric diagnoses in both sexes. Interaction effects between OC and CM were found for language, reading and writing problems in males. Vocabulary IQ emerged as a consistent protective factor.

**Conclusions:**

CM is a critical determinant of early psychopathology regardless child’s sex. OC contribute to neurodevelopmental and attentional problems only in males. Findings underscore the need for early identification and sex-specific interventions.

**Disclosure of Interest:**

None Declared

